# Bisphenol A Exposure during Pregnancy Alters the Mortality and Levels of Reproductive Hormones and Genes in Offspring Mice

**DOI:** 10.1155/2017/3585809

**Published:** 2017-03-14

**Authors:** Shuang Ma, Wanyu Shi, Xiaodan Wang, Pengyan Song, Xiuhui Zhong

**Affiliations:** ^1^College of Animal Science and Technology, Agricultural University of Hebei, Baoding 071001, China; ^2^College of Traditional Chinese Veterinary Medicine, Agricultural University of Hebei, Baoding 071001, China

## Abstract

The present study investigated the reproductive toxicity of bisphenol A (BPA) exposure to the mother on the offspring mice. BPA was given to pregnant mice at 50 mg/kg, 500 mg/kg, and 2500 mg/kg BW BPA daily by gavage during the whole gestation period. The offspring mice were sacrificed at 8 weeks of age. Results showed that exposure of BPA to the mother increased the mortality (*P* < 0.05). Maternal exposure of BPA reduced the levels of T (♂) and FSH (♀) (*P* < 0.01) and elevated E_2_ (♀) level in the adult offspring (*P* < 0.01). BPA exposure caused testicular damage as shown by less Leydig cells and ovarian injury as shown by more vacuoles and less corpus granules in the adult offspring mice. Immunohistochemistry revealed that maternal exposure of BPA increased Bax and decreased Bcl-2 at the protein levels in testicular and ovary tissues in the offspring mice. BPA significantly reduced the expression of StAR in male offspring (*P* < 0.05). Interestingly, the mRNA levels of Cyp11a were significantly decreased in 50 mg/kg groups and were increased in 500 mg/kg group in the males. Reduced Kitlg and elevated Amh at the mRNA levels were detected in the female offspring.

## 1. Introduction

Endocrine-disrupting compounds (EDCs) are the chemicals in the environment that can profoundly alter reproductive physiology and ultimately impact entire populations [1, 2]. EDCs have attracted great attention in the field of reproductive biology [3–5]. Bisphenol A (BPA) is one of the most studied EDCs and also one of the world's highest-production volume chemicals [5–7]. BPA is mainly used in the manufacture of polycarbonate plastics and epoxy resins. BPA can be found in a wide range of everyday products, such as food and drink packaging, the lining of aluminum food cans, and the coating of receipts, including food cans, bottle tops, and water supply pipes [5]. BPA is also found in polymers that are used in dental materials [8]. Exposure to BPA is not limited to humans, and effects on wildlife from contaminated water supplies have been documented in both males and females of multiple species [9, 10]. BPA exposure adversely affects the genetic and epigenetic integrity of mammalian oocytes [11].

The reproductive system is particularly susceptible to the endocrine-disrupting activity of BPA. BPA has been shown to interact with estrogen receptors and to act as an agonist or antagonist via estrogen receptor- (ER-) dependent signaling pathways. Therefore, BPA has been shown to play a key role in the pathogenesis of several endocrine disorders. Studies on variation in the timing, length, and dose of BPA exposure during pregnancy in animals have been conducted. These findings have identified that exposure to a very high concentration of BPA (100 mg/kg bw) during pregnancy adversely affects preimplantation embryo development, which in turn, completely inhibits implantation in mice [12], and decreases the number of live offspring rats [13]. Therefore, BPA poses significant public health concerns. So far, very little is known about the exact mechanisms of the action and pathological concentration of BPA as well as timing and length of BPA exposure that can negatively affect the metabolism and reproductive function of an individual [14].

The aim of the present study was to evaluate the developmental toxicity of BPA during the entire pregnancy on the offspring in mice, as well as its toxic effects on survival rate, the testicular or ovary structure, and reproductive genes.

## 2. Materials and Methods

### 2.1. Animals and Treatments

Eight-week-old female and male Kunming mice (of Clean Grade) were obtained from The SPFanimals Biotech Co. Ltd., Beijing, China. The mice were housed in polypropylene cages and maintained in temperature-controlled rooms at 23°C on a 12 h/12 h light-dark cycle. They were given food and water ad lib for 7-day acclimatization. Female mice were placed overnight with males. Detection of a vaginal plug in the next morning was designated as gestation day 0. Pregnant mice were randomly divided into 4 groups named A, B, C, and D, respectively. Group A was the control group. Mice in group A received normal ration and were gavaged with saline. Mice in groups B, C, and D were given BPA at 50 mg/kg, 500 mg/kg, or 2500 mg/kg body weight (>99% purity; Sigma-Aldrich Inc., USA) daily through gavage, respectively, from day 1 to the termination of pregnancy. A total of eighty pregnant mice (20 mice in each group) were included in the experiments. The use of animals of this study was approved by the Council for Animal Care in Hebei province.

Pups of all 4 groups were weaned at day 21 after birth and separated by sex. They were housed in temperature (23°C) and light-controlled (14-h light and 10-h dark) conditions [15] and received water and regular mouse chow ad lib. The female and male offspring were sacrificed by cervical dislocation at 8 weeks of age. Blood samples were obtained by puncturing the orbital vein, and the serum samples were separated by centrifuging at 3,000 ×g for 20 min in 4°C. The serum was stored at −80°C until assays. The intact testicular and ovarian tissues were carefully dissociated and removed, washed with PBS, and then weighed. The testicular index or ovary index was calculated as the ratio of testicular or ovary weights (mg) to the body weight (100 g). The left testicular and ovary tissues were fixed in Bouin's solution and embedded in paraffin for histological assays and hematoxylin-eosin (HE) staining. The left tissues were stored at −80°C for RT-PCR assays.

### 2.2. Serum Hormone Analysis

The serum estradiol (E_2_) level was analyzed with ELISA Kit (Beijing Reanta Biotech Co., Ltd., Beijing, China), Serum testosterone (T) levels were measured with Serum Testosterone ELISA Kit (Abcam, Cambridge, USA), Follicle stimulating hormones (FSH) were measured using an ELISA Kit (CUSABIO Life Science, MD, USA).

### 2.3. HE Staining and Immunohistochemistry (IHC)

The Bouin's solution-fixed, paraffin-embedded testicular or ovary specimens were cut into 5 *μ*m tissue sections. H&E staining was conducted routinely. For immunostaining, sections were incubated at 4°C overnight with a 1 : 500 dilution of the primary antibody Bax or Bcl-2. The Bax and Bcl-2 antibodies were purchased from Abcam (Cambridge, USA). The sections were then incubated at room temperature with the horseradish peroxidase- (HRP-) conjugated goat anti-rabbit IgG. Thirty minutes after the addition of the detection system, the reaction was visualized using diaminobenzidine (DAB) in the presence of hydrogen peroxide. The slides were examined using the Olympus IX71 Research Inverted Phase microscope (Olympus Co.), and the density was measured by Image J software (National Institute of Health, USA).

### 2.4. Reverse Transcription (RT) and Real-Time Quantitative PCR

Total RNA from the homogenates of the ovarian or testicular tissues was extracted using Trizol according to the instruction manual (Invitrogen, Carlsbad, USA). The RNA was reverse transcribed into cDNA using TaKaRa Super RT Kit accordingly (TaKaRa Biotechnology Co., Ltd., Dalian, China). The following substrates were added to the reaction tube, 2 *μ*g of RNA, 1 *μ*l of Oligo dT primer (50 *μ*M), 1 *μ*l of dNTP Mix, 4 *μ*l of 5x Reaction Buffer, 0.5 *μ*l of RNase Inhibitor (40 U/*μ*l), 1 *μ*l of MMLV RT (200 U/*μ*l), and 10.5 *μ*l of RNase-free dH_2_O for a total reaction volume of 20 *μ*l at 42°C for 45 min followed by 95°C for 5 min. The synthesized cDNA was stored at −80°C for subsequent real-time PCR analysis.

Real-time quantitative PCR was performed using the ABI Step-One Plus Real-Time PCR System (Applied Biosystems Inc., Foster City, CA, USA). *β*-Actin was used as the internal standard to control for the fluctuations of mRNA expression per sample. The first step of the amplification was a denaturation at 95°C for 5 min. The second step was a PCR reaction at 95°C for 15 s and 60°C for 30 s for a total of 40 cycles. The experiment was repeated four times. The relative mRNA expression levels of StAR, CYP11a, AMH, and Kitlg were calculated by setting the mRNA levels of *β*-actin as one. The results were presented as the mean ± standard error. The primer sequences that were used for mRNA expression detection are listed in Table 1.

### 2.5. Statistical Analysis

Data were recorded using the Excel database and further analyzed using SPSS19.0 software (IBM corporation, Armonk, NY, USA). Differences in parameters were analyzed by analysis of variance (ANOVA). The significance level were ^*∗*^*P* < 0.05 or ^*∗∗*^*P* < 0.01.

## 3. Results

### 3.1. Maternal Exposure to BPA Reduces the Survival Rate of the Offspring Mice

Results showed that the number of dead offspring in group C and group D on day 14 was 24 and 10, respectively. On day 21, there was 1 pup dead in group B, whereas those were 13 in group C and 1 in group D. There were 11 dead mice found in group D on day 42. Compared with the control group, the death rates in BPA groups were significantly increased (Table 2).

### 3.2. Maternal Exposure to BPA Affects the Reproductive Hormones of the Offspring Mice

The serum levels of T in the offspring mice of all BPA groups were reduced, which negatively correlated with the dosages of BPA (*P* < 0.01). The serum levels of FSH in mice of the BPA groups were significantly reduced compared with that of the control mice (*P* < 0.01). The serum levels of E_2_ in the offspring mice from each BPA group were significantly increased in a dose-dependent manner (*P* < 0.01; Table 3).

### 3.3. Organ Coefficients and Histology Changes in the Offspring Mice

As shown in Table 4, the testicular coefficient of the male offspring mice was decreased in the maternal BPA-exposed group (group B) only in comparison with the control mice (*P* < 0.05). No significant differences were observed in ovarian coefficients in BPA offspring compared with the control mice. There were no significant differences in body weights between the BPA-treated and control groups in both male and female offspring mice.

**Figure 1:**
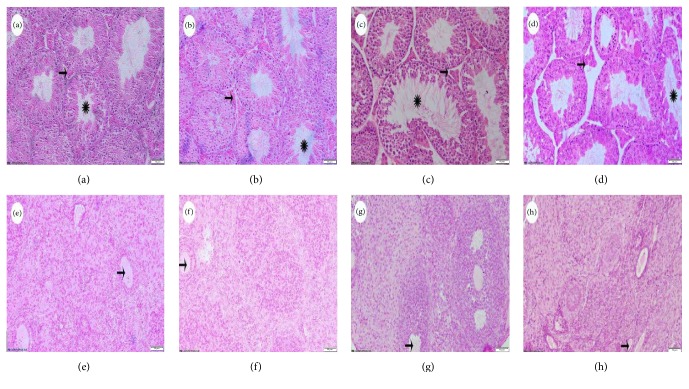
Organ histology changes of offspring mice (Bar = 50 *μ*m). (a) to (d) show testicular tissues: (a) control group; (b) 50 mg/kg BW BPA; (c) 500 mg/kg BW BPA; (d) 2500 mg/kg BW BPA. The Leydig cells were indicated by arrows; the seminiferous tubules were indicated by asterisks. (e) to (h) show the ovary: (e) control group; (f) 50 mg/kg BW BPA; (g) 500 mg/kg BW BPA; (h) 2500 mg/kg BW BPA. The ovarian vacuole was pointed by the arrow.

In contrast to the testicular (Figure 1(a)) or ovarian tissues (Figure 1(e)) from the control group, various degrees of histological abnormalities were observed in the BPA-treated groups. The testicular tissues exhibited irregular seminiferous epithelium with enlarged tubular lumen and thinner walls, immature germ cells sloughing into the tubular lumen, and Sertoli cell vacuolization in the BPA-treated groups. The interstitial cells became less in number compared to the normal structure.

Compared with the control group, more vacuoles were seen in the ovarian tissues with granular cell layer becoming lesser and disorderly arrayed in the BPA-treated offspring. Less follicles were detected and the microstructure of the ovary was obscure (Figure 1).

### 3.4. The Expressions of Bax and Bcl-2 in Testicular and Ovary Tissues

**Figure 2:**
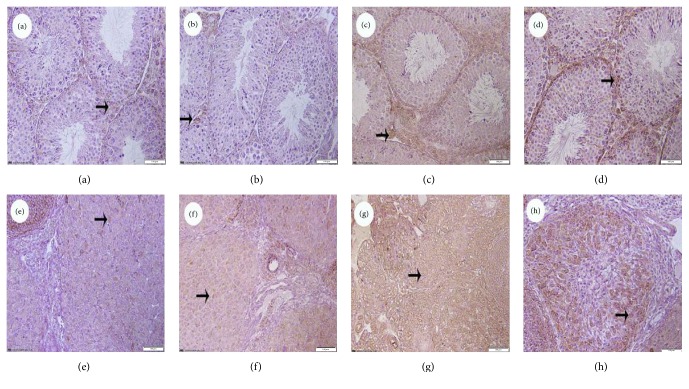
The expression of Bax in testicular and ovary tissues of offspring mice from BPA groups (Bar = 100 *μ*m). (a)–(d) show the testicular sections: (a) control group; (b) 50 mg/kg BW BPA; (c) 500 mg/kg BW BPA; (d) 2500 mg/kg BW BPA; (e)–(h) show the ovarian: (e) control group; (f) 50 mg/kg BW BPA; (g) 500 mg/kg BW BPA; (h) 2500 mg/kg BW BPA. Bax positive cells were indicated by arrows.

**Figure 3:**
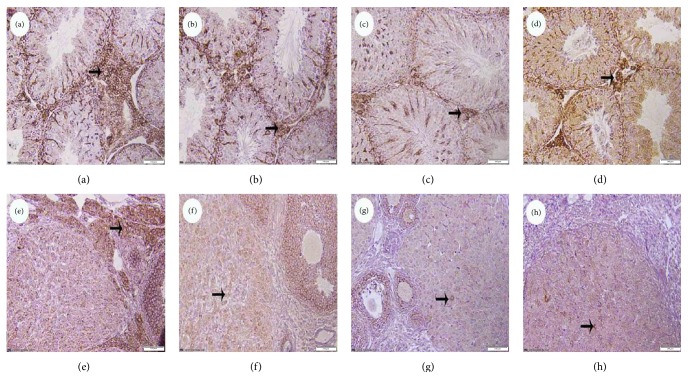
The expression of Bcl-2 in testicular and ovary tissues of offspring mice in BPA group (Bar = 100 *μ*m). (a)–(d) show the testicular tissues: (a) control group; (b) 50 mg/kg BW BPA; (c) 500 mg/kg BW BPA; (d) 2500 mg/kg BW BPA; (e)–(h) show the ovarian: (e) control group; (f) 50 mg/kg BW BPA; (g) 500 mg/kg BW BPA; (h) 2500 mg/kg BW BPA. Bcl-2 positive cells were pointed by arrows.

To investigate whether BPA decreased cell viability by inducing apoptosis, immunohistochemistry was performed. The positive cells were found in the Leydig cells or ovarian granular cells. Immunohistochemistry analysis of the testicular or ovary tissues of the offspring mice exposed to BPA were shown in Figures 2 and 3. The positive cells of Bax increased with the increasing doses of BPA (Figures 2(b), 2(c), 2(d), 2(f), 2(g), and 2(h)) and the expression of Bcl-2 decreased in BPA-treated groups (Figures 3(b), 3(c), 3(d), 3(f), 3(g), and 3(h)) compared with the control group.

### 3.5. Maternal BPA Exposure Affects Reproductive Genes in Testis or Ovaries of the Offspring Mice

Real-time PCR revealed that the mRNA levels of StAR in BPA groups were decreased (*P* < 0.05) in comparison with the control. The mRNA levels of CYP11a gene in groups of B and D were significantly decreased (*P* < 0.01) compared with the control, while the levels of CYP11a in group C were significantly increased (*P* < 0.01). The mRNA expression levels of the AMH gene in the groups C and D were significantly increased (*P* < 0.01). The mRNA expression levels of the Kitlg gene in groups C and D were lower than that of the control group (Table 5).

## 4. Discussions

Bisphenol A (BPA) as a ubiquitous environmental endocrine disruptor is present in polycarbonate plastics, epoxy resins, paper receipts, and cardboards [16, 17]. Exposure to BPA is not limited to humans, but also to other animals of multiple species [9, 10]. Animal studies have shown that developmental BPA exposure results in a wide range of adverse effects, including reproductive, cardiovascular, immunological, metabolic, behavioral, and neurological disorders as well as certain cancers in adult offspring [18–20]. Developmental BPA exposure modifies Fkbp5 methylation and impacts stress responsiveness [21]. Reports from in vitro studies indicate BPA exposure affects early embryo development [12, 14].

In the present study, we demonstrate that prenatal exposure to BPA results in increased death rate, elevated E_2_ levels, and Bax protein expressions and AMH gene expressions in both male and female adult offspring. Meanwhile, our results also indicate that BPA exposure during pregnancy downregulates FSH, T levels, and Bcl-2 protein and the expressions of StAR gene in adult offspring. Thus, the present findings provide a novel insight into the long-term and sex-specific effects of developmental BPA exposure on reproductive development.

The concentrations of BPA present in the environment vary greatly. The highest amounts of BPA (up to 17 mg/L) are reported in landfill leachate and pulp mill effluents [22], whereas in river and marine sediments 43 and up to 191 mg/kg dry weight were reported [22, 23]. In animal studies, oral exposure of BPA at 500 mg/kg daily from gestation day 6 to postnatal day 21 affects the behavioral development of rat offspring [24]. BPA exposure to 20-day-old zebrafish fry at a dose of 2000 mg/kg diet daily for 45 days induced feminization of the fry [25]. The dosages of BPA determined in our present study were based on our previous dose-response studies as well as on others' reports.

At the first step in investigating the effects of prenatal BPA exposure on the pups, we found that the survival rate was significantly reduced in both male and female mice prenatally exposed to BPA when compared to the offspring of the control mice. BPA did not alter body weight in both male and female mice (Table 4). A similar result in the ratio of ovary weight to body weight in prenatally BPA-exposed offspring was observed. BPA exposure to pregnant mice did not alter the body weight of the offspring mice [18]. This is in marked contrast to the report [26] in which maternal BPA exposure during pregnancy and lactation led to increased body weight and visceral adipose tissue in both female and male offspring rats. Ma et al. also observed elevation in body weight in the offspring rats in BPA-treated groups [27]. Although the precise reasons for the discrepancy between our study and the others are not clear, it might be that differences in the dosage of BPA and the length of its exposure as well as the animal species are important contributing factors.

An important physiological function of the ovary is to synthesize estrogen, whereas the Leydig cells in testis are to synthesize testosterone (T). Studies have indicated that the levels of BPA in urine were negatively correlated with the level of serum E_2_ in infertile women [28]. BPA and titanium dioxide nanoparticles coexposure caused a reduction in plasma concentrations of E_2_, T, and FSH [29]. Ma et al. [27] reported that maternal exposure to BPA by gavage at 250 mg/kg daily reduced T, LH, FSH, and E_2_ levels in the female offspring rats. Lee et al. [30] also observed exposure to BPA significantly decreased E_2_ serum concentration in rats. While a positive correlation between urine BPA and E_2_ was observed among BPA-exposed women workers [31]. In contrast to previous reports, our present study revealed an increased serum concentration of E_2_ in offspring female mice of the maternal BPA-exposed groups. The increased concentration of this estrogen indicates the estrogenic effect of BPA. This finding points to a hormonal imbalance that can cause disorders in histopathological changes in the testis and ovary [32]. Regulation of reproductive processes occurs directly through the influence of T and indirectly through its metabolites, DHT and E_2_. A high concentration of E_2_ disrupts spermatogenesis and steroidogenesis, stimulating Leydig cell hyperplasia and reducing androgen synthesis by inhibiting the activities of enzymes involved in the synthesis of T [33].

To assess the relationship of apoptosis in the testis and ovary to BPA exposure in offspring mice, we investigated the protein expression levels of Bcl-2 and Bax, which belong to the Bcl-2 family. Bcl-2 inhibits, and Bax promotes apoptosis by affecting the mitochondrial membrane permeability and cytochrome C release [34, 35]. Ovarian malformations including increased number of blood-filled ovarian bursae and decreased corpora lutea have also been observed. The results from our study of the protein expression analysis revealed decreased Bcl-2 and increased Bax levels in BPA exposure-induced apoptosis in spermatogenic cells and granulosa cells, suggesting that BPA affects reproductive system via the mitochondrial apoptotic pathway. In addition, the altered histology of the reproductive organ and downregulation of Kitlg expression observed in the BPA-exposed groups may also be associated with the protein expression levels of Bcl-2 and Bax.

Testosterone is synthesized from cholesterol, which is stored in cytoplasmic lipid droplets that can be synthesized de novo from acetyl-coenzyme within the Leydig cells or that can be uptaken by cells through endocytosis of low-density lipoproteins [36]. In the Leydig cells, the steroidogenic acute regulatory protein (StAR) transports cholesterol from the outer to the inner mitochondrial membrane. Our results reveal that the transcript level of StAR in BPA-treated male offspring is significantly reduced compared with the one observed in the control group (Table 5). This is in agreement with the results of Lee and his colleagues [30], who reported the decreased expression of StAR after BPA administration. A plausible explanation for this phenomenon is the alteration of the posttranslational phosphorylation of StAR which regulates its activity. It is well known that CYP11a is the rate-limiting enzyme for testosterone synthesis in Leydig cells [37]. Results of our present study indicate that BPA exposure during the whole pregnant period downregulates CYP11a expression (Table 5), suggesting that BPA exposure may decrease testosterone production via downregulation of CYP11a. In addition, the changed histology of testis caused by BPA may also be associated with the downregulation of StAR and CYP11a expressions.

Kitlg has been previously reported to be essential to ovarian development [38] and is an important element in inhibiting apoptosis in different cell types [39]. Our results have shown that BPA downregulates Kitlg expression (Table 5), which may increase the protein level of Bax in offspring mice, suggesting that BPA exposure has inhibitory effects on the development and maturation of oocytes and follicles by interfering with the expressions of Bax and Bcl-2. AMH is a negative regulatory factor in follicular growth and development. It comes from developing follicles and is not expressed in primordial follicles, as its presence could block follicular development [40, 41]. We have observed that BPA upregulates AMH gene expression (Table 5), suggesting that BPA may induce the upregulation of the AMH gene and inhibit follicular development through an increase of AMH levels. In addition, the morphological alteration of the ovarian caused by BPA may also be associated with the downregulation of Kitlg expression and the upregulation of AMH expression.

In summary, our results indicate that BPA exposure during pregnancy has deleterious effects on testicular and ovarian developments and their functions of the offspring mice, which were observed as developmental disorders in the sex hormone secretion. The downregulation of Bcl-2 and StAR and CYP11a gene expressions and the upregulation of AMH gene expression and Bax may play certain roles in the pathogenesis of BPA-induced toxicity during offspring mice development.

## Figures and Tables

**Table 1:** Sequences of the primers in RT-PCR.

Genes	Sequences
StAR	F: TCAGCATGTTCCTCGCTACG
	R: CCTGCTGGATGTAGGACAGC
CYP11a	F: CCAACCTTTCCTGAGCCCTAC
	R: AAACTGACTCCAAAGTGCCCA
Kitlg	F: ATTGTCAACGTGGACCAGTG
	R: CGCTAGAGTGACTGTCAAGG
AMH	F: TGGTGACAGTGAGAGGAGAG
	R: CAGGAAGGCATACTCATAGC
*β*-Actin	F: GCAGATGTGGATCAGCAAGC
	R: AGGGTGTAAAACGCAGCTCAG

**Table 2:** Maternal exposure to BPA reduces the survival rate of the offspring mice.

Groups	Day 7	Day 14	Day 21	Day 28	Day 35	Day 42	Day 49	Day 56	Death rate
A (*n* = 114)	0	0	0	1	0	0	0	0	0.8 (1/114)
B (*n* = 105)	0	0	1	1	0	2	0	0	3.8 (4/105)^*∗∗*^
C (*n* = 98)	0	24	13	1	0	0	0	0	38.8 (38/98)^*∗∗*^
D (*n* = 101 )	0	10	1	3	0	11	0	0	24.8 (25/101)^*∗∗*^

*Note*. Group A is the control. Group B is the BPA low dose group (50 mg/kg), group C is the 500 mg/kg group, and group D is the 2500 mg/kg group. *∗∗* means *P* < 0.01.

**Table 3:** Effect of BPA on reproductive hormone of offspring mice.

Groups	T	FSH	E_2_
A	2.86 ± 0.18^C^	9.52 ± 0.05^C^	47.77 ± 0.13^A^
B	1.40 ± 0.32^B^	5.56 ± 0.05^A^	56.84 ± 0.12^B^
C	0.06 ± 0.01^A^	6.37 ± 0.55^B^	59.45 ± 0.20^C^
D	0.04 ± 0.01^A^	5.47 ± 0.09^A^	90.92 ± 0.20^D^

Group A is the control group. Group B is the BPA low dose group (50 mg/kg). Group C is the 500 mg/kg group. Group D is the 2500 mg/kg group. Different capital letters indicate significance (*P* < 0.01).

**Table 4:** Testicular and ovarian weight and organ coefficient changes in the offspring mice.

Group	*n*	♂ body weight/g	Testicular weight/g	Testicular coefficient/%	♀ body weight/g	Ovarian weight/g	Ovarian coefficient/%
A	20	30.10 ± 1.19	0.24 ± 0.01^b^	0.82 ± 0.03^b^	29.51 ± 1.27	0.03 ± 0.004	0.08 ± 0.01
B	20	29.75 ± 0.70	0.20 ± 0.01^a^	0.68 ± 0.02^a^	27.22 ± 0.36	0.04 ± 0.014	0.17 ± 0.05
C	20	28.49 ± 1.56	0.21 ± 0.02^a^	0.74 ± 0.07^ab^	26.56 ± 0.40	0.06 ± 0.021	0.24 ± 0.08
D	20	28.85 ± 1.06	0.21 ± 0.01^a^	0.74 ± 0.02^ab^	30.16 ± 0.75	0.08 ± 0.024	0.27 ± 0.09

Group A is the control group. Group B is the BPA low dose group (50 mg/kg). Group C is the 500 mg/kg group. Group D is the 2500 mg/kg group. Different capital letters indicate significance (*P* < 0.01).

**Table 5:** Relative mRNA expressions of the offspring mice in BPA groups.

Groups	StAR	CYP11a	AMH	Kitlg
A	1.00 ± 0.016^b^	1.00 ± 0.022^C^	1.00 ± 0.020^A^	1.00 ± 0.041^B^
B	0.35 ± 0.05^a^	0.35 ± 0.004^B^	0.48 ± 0.007^A^	0.94 ± 0.012^B^
C	0.54 ± 0.027^a^	1.84 ± 0.035^D^	9.09 ± 0.270^C^	0.58 ± 0.029^A^
D	0.51 ± 0.058^a^	0.15 ± 0.005^A^	6.70 ± 0.355^B^	0.70 ± 0.017^A^

Group A is the control group. Group B is the BPA low dose group (50 mg/kg). Group C is the 500 mg/kg group. Group D is the 2500 mg/kg group. Different capital letters indicate significance (*P* < 0.01).
